# Citation Contamination by Paper Mill Articles in Systematic Reviews of the Life Sciences

**DOI:** 10.1001/jamanetworkopen.2025.15160

**Published:** 2025-06-12

**Authors:** Gengyan Tang, Hao Cai

**Affiliations:** 1 Werklund School of Education, University of Calgary, Calgary, Alberta, Canada; 2 The First Affiliated Hospital, Chongqing Medical University, Chongqing, China

## Abstract

**Importance:**

Systematic reviews are the criterion standard for evidence synthesis in the life sciences, yet their reliability and integrity are threatened by citation contamination from fabricated publications produced by paper mills. Despite growing awareness, the extent and implications of this issue remain unclear.

**Objectives:**

To analyze the prevalence, characteristics, affected subject areas, and citation patterns of retracted paper mill articles cited in systematic reviews.

**Design, Setting, and Participants:**

This cross-sectional study analyzed systematic reviews published between 2013 and 2024, indexed in Web of Science (WoS). References were matched against the Retraction Watch dataset, and full texts were reviewed to identify retracted paper mill articles incorporated into the evidence synthesis.

**Main Outcomes and Measures:**

The study assessed (1) contamination prevalence, defined as the proportion of systematic reviews incorporating retracted paper mill articles into the evidence synthesis; (2) geographic distribution of citing authors according to institutional affiliations; (3) citation timing and trends, including the time lag between incorporation and article retraction; (4) affected research areas, categorized by WoS subject classifications; and (5) citation patterns, including highly contaminated reviews (≥3 incorporations of retracted articles).

**Results:**

Of the total of 200 000 systematic reviews, 299 incorporated at least 1 retracted paper mill article into the evidence synthesis (contamination rate, 0.15%). Among them, 256 (85.6%) included a single retracted article, and 43 (14.4%) included multiple such articles. Of 1802 author affiliations associated with the contaminated reviews, 660 (36.6%) were from institutions in China. Of 385 total citations, 124 (32.2%) occurred after retraction, including 13 occurring more than 500 days after the retraction date. Oncology was the most affected field (48 of 299 [16.1%]). Five reviews each included 5 or more retracted articles, all published in journals under questionable publishers.

**Conclusions and Relevance:**

In this cross-sectional study of life sciences systematic reviews, contamination remained low but increased over time, posing a risk to research integrity. Continued citation of retracted articles, even after retraction, highlights the need for rigorous screening practices. Correcting contaminated reviews and developing automated detection tools are essential to preserving the credibility of systematic reviews.

## Key Points

**Question:**

Do paper mill articles contaminate systematic reviews in the life sciences, and what are their implications for the integrity of evidence synthesis?

**Findings:**

In a cross-sectional study of 200 000 systematic reviews published between 2013 and 2024, 0.15% incorporated retracted paper mill articles into the evidence synthesis, with an increase over time. Oncology was the most affected field, and a total of 124 citations occurred after retraction, including 13 occurring more than 500 days after the retraction date.

**Meaning:**

These findings highlight the growing risk of citation contamination in systematic reviews and underscore the need for enhanced screening measures, systematic correction of contaminated reviews, and automated detection tools to protect the integrity of evidence synthesis.

## Teaser

This cross-sectional study analyzes the prevalence, characteristics, affected subject areas, and citation patterns of retracted paper mill articles cited in systematic reviews.

## Introduction

Systematic reviews are widely regarded as the criterion standard for synthesizing high-quality evidence in the life sciences by integrating findings from multiple studies to generate aggregated conclusions and identify research gaps.^1,2,3,4^ Their structured method aims to minimize bias, yet concerns remain regarding the reliability of the evidence they incorporate. Factors such as the preferential inclusion of positive results, the exclusion of gray literature, and language restrictions can distort their findings.^5,6,7,8,9,10,11,12^ Although frameworks such as PRISMA 2020 help mitigate bias in systematic reviews, they cannot fully safeguard against a growing and more insidious threat: the infiltration of fraudulent publications from paper mills.^13^

Paper mills, commercial enterprises that fabricate scientific manuscripts and sell authorship positions, have been identified as a major challenge to research integrity.^14^ Unlike isolated cases of misconduct, these operations produce large volumes of systematically falsified studies, often containing manipulated images, fabricated data, and citation manipulation.^15,16,17^ Some of these articles have been flagged in systematic reviews on stroke treatments, raising concerns about the integrity of evidence synthesis.^18^ Despite increasing awareness, empirical research on how such fabricated studies contaminate systematic reviews remains scarce. In November 2024, the United2Act group, supported by COPE (Committee on Publication Ethics) and STM (International Association of Scientific, Technical & Medical Publishers), released a consensus statement urging further investigation into the outcomes of paper mills on scientific literature, emphasizing that current understanding is largely anecdotal.^19^

Although previous studies have characterized the methods of paper mills and their distinct features,^20,21,22^ little is known about their downstream effects on evidence-based research. Given that systematic reviews directly inform clinical decision-making,^23^ the presence of fraudulent citations could have profound implications for patient care and policy recommendations. This study systematically examines the extent of citation contamination in systematic reviews indexed in the Web of Science (WoS), assessing (1) the contamination prevalence, (2) their geographic distribution, (3) citation timing and trends, (4) research area distributions, and (5) citation patterns. The findings highlight the extent to which paper mills have infiltrated the scientific literature, raising urgent concerns about the integrity of synthesized evidence in both academic and clinical contexts.

## Methods

This cross-sectional study examines systematic review publications in the life sciences and adheres to the Strengthening the Reporting of Observational Studies in Epidemiology (STROBE) reporting guidelines. Because the study does not involve human participants or animal subjects and relies solely on publicly available published data, institutional review board approval and informed consent were not required, in accordance with 45 CFR §46.

### Data Management and Analysis Tools

Data management, categorization, and filtering were conducted using Microsoft Excel version 2403. Citation matching was conducted using Python version 3.9.10.

### Data Acquisition and Filtering

Four datasets were used in this study. The first was the Retraction Watch dataset.^24^ Bibliographic data on 61 932 retracted publications, including publication dates, retraction dates, DOIs, and retraction reasons, were obtained from the Retraction Watch dataset. Entries labeled *paper mills* were filtered, resulting in 10 409 retracted publications used for citation matching.

The second dataset used was WoS.^25^ Systematic reviews published between 2013 and 2024 in the Life Sciences and Biomedicine category were retrieved from the WoS Core Collection via a title-based search. After initial filtering, 240 170 publications met inclusion criteria. Because of database limits, the top publications by relevance were analyzed. Metadata extracted included publication dates, journal names, DOIs, authors, and cited references for citation matching. The detailed search strategy is provided in eFigure 1 in Supplement 1.

The third dataset used was OpenAlex. Geographic locations of authors citing retracted paper mill articles were identified using institutional affiliations from OpenAlex via article DOIs. For authors with multiple affiliations, the first-listed institution was used. OpenAlex data informed geographic distribution analysis.

The fourth dataset was the Altmetric database. Citations of highly contaminated systematic reviews (≥3 retracted citations) were retrieved from Altmetric.^26^ These data informed analyses of citation patterns and dissemination beyond academia.

### Detection of Paper Mill Citations in Systematic Reviews

A Python script (detailed in eAppendix 1 in Supplement 1) was used to extract bibliographic data from the WoS database, including article titles, authors, journal titles, publication years, references, and research areas. If multiple research areas were assigned to a publication, only the first-listed category was considered. Cited reference DOIs were matched against the Retraction Watch dataset to identify citations of retracted publications, with metadata on publication and retraction dates extracted for citation timing analysis.

Citation matching identified 630 citations across 524 systematic reviews. Twelve invalid citations, each corresponding to a unique systematic review, were excluded because of database entry errors, leaving 618 citations within 512 systematic reviews. A full-text review (detailed in eAppendix 2 in Supplement 1) assessed whether these citations were incorporated into evidence synthesis, as such citations are most likely to influence review conclusions. Systematic reviews were classified as contaminated if they incorporated at least 1 retracted paper mill article into the evidence synthesis, defined as (1) inclusion in a meta-analysis or (2) citation as supporting evidence in the results section of a narrative systematic review. The final dataset was structured and saved as a CSV file for analysis.

### Statistical Analysis

Descriptive statistics were used to assess contamination prevalence, geographic distribution of citing authors, citation timing and trends, research area patterns, citation patterns, and journal susceptibility. Contamination prevalence was calculated as the proportion of systematic reviews citing retracted paper mill publications relative to the total identified. Geographic distribution was analyzed by aggregating institutional affiliations from OpenAlex and determining the number of unique countries and continents represented.

Citation timing was assessed by calculating the time lag between the systematic review publication date and the retraction date of cited articles, with negative values indicating preretraction citations and positive values indicating postretraction citations. Annual citation trends were analyzed on the basis of the number of systematic reviews published each year that cited retracted paper mill articles and the total citation count within systematic reviews. Systematic reviews were classified into research areas according to WoS subject categories. Citation patterns were examined by identifying systematic reviews citing 3 or more retracted articles. All analyses were performed in R statistical software version 4.4.2 (R Project for Statistical Computing) using the dplyr package.

## Results

### Contamination Prevalence

Of the total of 200 000 articles, 299 systematic reviews in the life sciences were identified as incorporating paper mill articles in evidence synthesis, for a contamination rate of 0.15%. These reviews collectively cited paper mill articles 385 times. Among them, 256 of 299 (85.6%) cited only a single retracted paper mill article, whereas 43 of 299 (14.4%) cited 2 or more retracted articles linked to paper mills. Two of 299 (0.7%) cited 6 paper mill articles each, and 1 of 299 (0.3%) cited 13 such articles.

### Geographic Distribution of Authors Citing Paper Mill Articles

Among the systematic reviews in the life sciences citing paper mill articles, 1802 authors affiliated with institutions across 52 countries were identified (Table). At the national level, the largest proportion of these authors were affiliated with institutions in China (660 of 1802 [36.6%]), followed by the US (139 of 1802 [7.7%]), Iran (134 of 1802 [7.4%]), Italy (133 of 1802 [7.4%]), and India (66 of 1802 [3.7%]). This distribution suggests that systematic reviews from institutions in certain regions may be more likely to cite paper mill articles.

**Table.** Global Distribution of Authors Citing Paper Mill Articles

Continent and country	Authors, No. (% of total) [% within continent] (N = 1802)
Asia (n = 1013)	
China	660 (36.63) [65.15]
Iran	134 (7.44) [13.23]
India	66 (3.66) [6.52]
South Korea	39 (2.16) [3.85]
Saudi Arabia	28 (1.55) [2.76]
Vietnam	21 (1.17) [2.07]
Malaysia	19 (1.05) [1.88]
Japan	15 (0.83) [1.48]
Indonesia	8 (0.44) [0.79]
Pakistan	6 (0.33) [0.59]
Iraq	5 (0.28) [0.49]
Thailand	4 (0.22) [0.39]
United Arab Emirates	4 (0.22) [0.39]
Sri Lanka	1 (0.06) [0.10]
Syria	1 (0.06) [0.10]
Jordan	1 (0.06) [0.10]
Singapore	1 (0.06) [0.10]
Europe (n = 488)	
Italy	133 (7.38) [27.25]
United Kingdom	63 (3.50) [12.91]
Spain	51 (2.83) [10.45]
Poland	32 (1.78) [6.56]
Romania	27 (1.50) [5.53]
Russia	24 (1.33) [4.92]
Germany	23 (1.28) [4.71]
Portugal	20 (1.11) [4.10]
Switzerland	19 (1.05) [3.89]
Greece	18 (1.00) [3.69]
Netherlands	16 (0.89) [3.28]
France	10 (0.55) [2.05]
Serbia	10 (0.55) [2.05]
Ireland	10 (0.55) [2.05]
Belgium	8 (0.44) [1.64]
Sweden	6 (0.33) [1.23]
Norway	6 (0.33) [1.23]
Denmark	4 (0.22) [0.82]
Austria	3 (0.17) [0.61]
Bulgaria	2 (0.11) [0.41]
Turkey	1 (0.06) [0.20]
Bosnia and Herzegovina	1 (0.06) [0.20]
Hungary	1 (0.06) [0.20]
North America (n = 178)	
US	139 (7.71) [78.09]
Canada	28 (1.55) [15.73]
Mexico	11 (0.61) [6.18]
Australia (n = 23)	23 (1.28) [100.00]
South America (n = 63)	
Brazil	47 (2.61) [74.6]
Colombia	15 (0.83) [23.81]
Peru	1 (0.06) [1.59]
Africa (n = 37)	
Egypt	17 (0.94) [45.95]
Ethiopia	10 (0.55) [27.03]
Morocco	5 (0.28) [13.51]
Ghana	4 (0.22) [10.81]
Libya	1 (0.06) [2.7]

At the continental level, most authors were affiliated with institutions in Asia (1013 of 1802 [56.2%]), followed by Europe (488 of 1802 [27.1%]), North America (178 of 1802 [9.9%]), South America (63 of 1802 [3.5%]), Africa (37 of 1802 [2.1%]), and Australia (23 of 1802 [1.3%]). The predominance of Asian institutions, particularly those in China (660 of 1013 [65.2%]), may reflect regional differences in the frequency of citations to paper mill articles.

### Citation Timing and Trends

**Figure 1.**
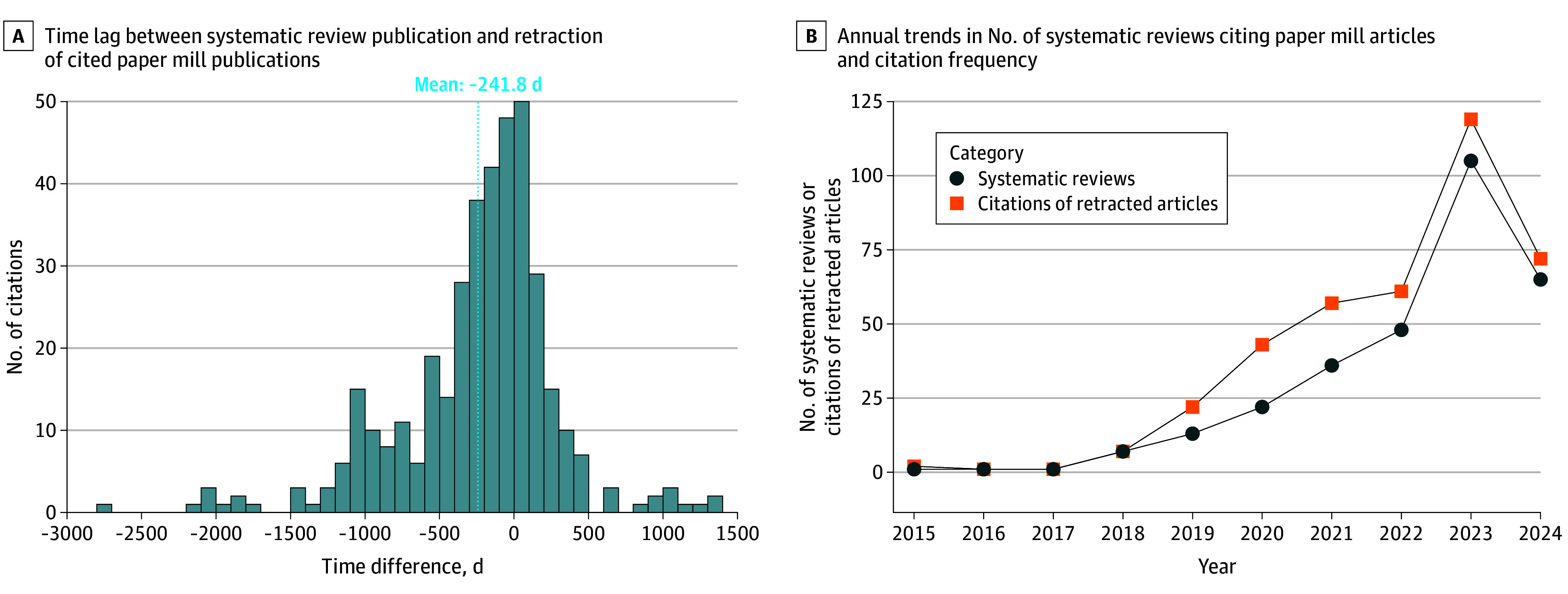
Temporal Patterns of Citation to Retracted Paper Mill Articles Graphs show time lag between systematic review publication and retraction of cited paper mill publications (A) and annual trends in the number of systematic reviews citing paper mill articles and citation frequency (B).

The analysis of citation time lags (Figure 1A) indicates that most citations to paper mill articles occurred before their retraction, with 224 of 385 (58.2%) occurring within 1000 days before retraction. However, 124 of 385 (32.2%) were made after the articles had been retracted, including 13 of 385 (3.4%) that occurred more than 500 days after retraction. Citations ranged from 2773 days before to 1306 days after retraction, with a mean of 241.8 days before retraction, suggesting that many systematic reviews referenced these articles prior to their removal, although postretraction citations remained notable.

A substantial increase was observed in both the number of systematic reviews in the life sciences citing paper mill articles and the total citations to these articles (Figure 1B). No such reviews were identified in 2013 or 2014. The number of systematic reviews increased from 1 in 2015 to a peak of 105 in 2023, before declining to 65 in 2024. A similar trend was observed for citations, which increased from 2 in 2015 to 119 in 2023, and then declined to 72 in 2024. These findings suggest an overall increase until 2023, followed by a decline that may be influenced by retraction delays and the citation of newer articles, some of which may not yet have been identified as paper mill publications or retracted.

### Research Area Distributions

**Figure 2.**
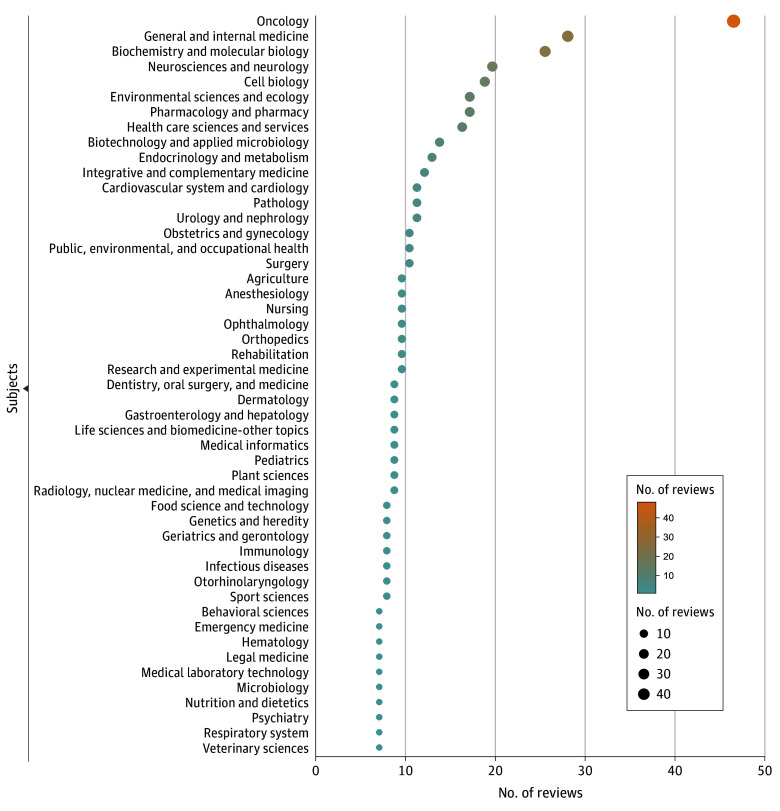
Research Areas Affected by Paper Mill Contamination in Systematic Reviews Bubble chart shows the number of systematic reviews citing retracted paper mill articles across research areas in the life sciences. Each bubble represents 1 subject area, with size and color indicating the number of contaminated reviews. Oncology had the highest count, followed by general and internal medicine, biochemistry and molecular biology, and neurosciences and neurology.

Among the 76 research areas classified under the life sciences and biomedicine category in the WoS, 52 (68.4%) included systematic reviews that cited retracted articles linked to paper mills, although the number of contaminated reviews varied by area. As shown in Figure 2, oncology was the most affected field, accounting for 48 of 299 contaminated systematic reviews (16.1%). This was followed by general and internal medicine (26 of 299 [8.7%]), biochemistry and molecular biology (23 of 299 [7.7%]), neurosciences and neurology (16 of 299 [5.4%]), and cell biology (15 of 299 [5.0%]). Research areas with 10 to 14 contaminated reviews included environmental sciences and ecology (13 of 299 [4.3%]), pharmacology and pharmacy (13 of 299 [4.3%]), and health care sciences and services (12 of 299 [4.0%]).

### Citation Patterns

Among the 17 of 299 (5.7%) systematic reviews classified as heavily citation-contaminated (ie, citing paper mill articles 3 or more times), 5 of 17 reviews (29.4%)^27,28,29,30,31^ each cited 5 or more paper mill articles (shown as SY2, SY3, SY4, SY5, and SY11 in eFigure 2 and eTable in Supplement 1). Notably, Hitu et al^29^ referenced 13 retracted paper mill articles. Wu et al^30^ (1 of 5 [20.0%]) was published in a Frontiers Media S.A. journal, and the remaining 4 (80.0%) appeared in journals published by the Multidisciplinary Digital Publishing Institute. These reviews incorporated fabricated evidence into their syntheses, potentially affecting the validity of their conclusions. According to Altmetric, Favier et al^27^ has been cited in 23 publications, Maiese et al^28^ in 16, Hitu et al^29^ in 20, Wu et al^30^ in 20, and Klicka et al^31^ in 23. Furthermore, Favier et al^27^ was cited in a rule published by the US federal government on January 18, 2023. This rule added all types of uterine cancer, including endometrial cancer, to the list of World Trade Center–related health conditions.^32^

## Discussion

Systematic reviews play a crucial role in the life sciences by synthesizing high-quality evidence to inform clinical practice and guide future research.^33^ Consequently, the integrity and reliability of the literature analyzed in systematic reviews are essential.^34^ Although numerous studies have raised concerns about the methodological quality of studies included in systematic review,^35,36,37,38,39^ few have specifically examined the integrity of the evidence incorporated in these reviews. The large-scale retraction of publications linked to paper mills has heightened awareness of fabricated research within the academic community.^40,41,42^ However, limited studies have assessed the extent to which these retracted articles have infiltrated systematic reviews, despite the fundamental need for high-quality evidence in these analyses. By systematically examining the references cited in systematic reviews, this cross-sectional study provides the first, to our knowledge, comprehensive investigation of this issue, offering novel insights into the outcomes of paper mill articles in evidence synthesis.

We conducted a large-scale analysis of the references cited in 200 000 life sciences systematic reviews indexed in the WoS database. These references were cross-matched with retracted articles recorded in the Retraction Watch dataset that were linked to paper mills. For all systematic reviews that cited such retracted articles, we performed full-text reviewing to identify whether the retracted articles were incorporated into the evidence synthesis. Reviews that included paper mill articles in their synthesis were classified as citation contaminated. Our findings indicate that although the overall citation contamination of life sciences systematic reviews by paper mill articles remains low (contamination rate, 0.15%), the number of contaminated citations appears to be gradually increasing. This trend aligns with the increasing number of retractions associated with paper mills.^43^ We also observed that the majority of authors of citation-contaminated systematic reviews were affiliated with institutions in China, which may be linked to the country’s frequent paper mill activities. This finding is consistent with previous studies on paper mill–related publications.^44^ However, unlike prior research, we also identified authors of contaminated systematic reviews from institutions in countries outside Asia, including the US, Italy, Brazil, Canada, and the United Kingdom. This observation suggests that citation contamination in systematic reviews may be emerging as a global issue.

Our further analysis indicates that the retraction of paper mill articles does not necessarily mark the end of their citation life cycle. We identified 124 instances in which systematic reviews cited paper mill articles after their retraction, suggesting that the authors did not consistently verify the retraction status of the studies they cited. This finding aligns with previous reports.^45,46^ A more challenging issue is that many citations occurred before the retraction of the referenced articles, posing difficulties for the correction of systematic reviews. Our analysis suggests that citation contamination in systematic reviews by paper mill articles is concentrated in specific research fields, with oncology, general and internal medicine, biochemistry and molecular biology, and neurosciences and neurology being the most affected. Previous studies have identified oncology and molecular biology as heavily impacted by retractions.^47^ Our findings extend this conclusion, indicating that contamination may also affect multiple other disciplines.

We also analyzed heavily contaminated systematic reviews, defined as those citing 3 or more retracted paper mill articles. These reviews exhibited similar citation patterns, with the cited paper mill articles predominantly appearing in the results section. Among these contaminated systematic reviews, the 5 most frequently citing reviews were published in journals associated with questionable academic publishers.^48^ Previous studies have reported excessively high self-citation rates in journals from these publishers.^49^ Our findings further suggest that systematic reviews published in these venues may lack adequate consideration of evidence quality. In addition, all 5 of these systematic reviews had been cited more than 10 times, with one even referenced in a government regulation related to life sciences. This finding underscores the potential influence of contaminated systematic reviews on subsequent research and policymaking, warranting heightened scrutiny from the academic community.

Given the emphasis on high-quality evidence in systematic reviews and their critical role in the life sciences, we believe it is essential to issue corrections or retraction notices for systematic reviews that have cited retracted paper mill articles. Academic journals may need to reassess the reliability of these reviews to ensure the validity of their conclusions. For systematic reviews heavily affected by citation contamination, journals should also consider whether these reviews themselves may be products of paper mills. In addition, we recommend that journals implement citation-screening tools during the review process to detect references to articles retracted owing to paper mill activity. Ultimately, a coordinated effort from the academic community is required to mitigate the spread of citation contamination in systematic reviews.

### Limitations

Our cross-sectional study has several limitations. We could only match systematic reviews to identified and retracted paper mill articles, but many undiscovered cases likely remain. Thus, our analysis may represent only a fraction of the true extent of contamination, potentially underestimating the citation prevalence in life sciences systematic reviews. Database restrictions prevented the evaluation of some publications, introducing possible selection bias and further underestimation of contamination rates. In addition, the strict inclusion criteria of WoS result in fewer indexed publications than other databases, which may also contribute to underestimation. Furthermore, we did not assess whether cited paper mill articles influenced the findings or conclusions of systematic reviews. Future research should explore this aspect.

## Conclusions

In this cross-sectional study of life sciences systematic reviews, we identified instances in which retracted articles from paper mills were incorporated into the evidence synthesis. Although the overall number of contaminated reviews was low, their frequency increased over time. The inclusion of fabricated studies in evidence syntheses may compromise the integrity and validity of systematic reviews and introduce bias into downstream research and clinical practice. These findings highlight the need for rigorous screening of included studies to ensure the integrity of systematic review evidence.

## Notes

Supplement 1. **eFigure 1.** Flowchart of Data Screening **eFigure 2.** Citation Network of Systematic Reviews with Heavy Citation Contamination **eTable.** DOIs of Systematic Reviews with Severe Citation Contamination and the DOIs of Their Cited Papers **eAppendix 1.** Python Source Code for Data Matching **eAppendix 2.** Full-Text Review Strategy

Supplement 2.
Data Sharing Statement
