# Supplementary material for: Citation Contamination by Paper Mill Articles in Systematic Reviews of the Life Sciences
Source: JAMA Netw Open. 2025 Jun 12;8(6):e2515160. doi: 10.1001/jamanetworkopen.2025.15160 (PMC12163679; doi:10.1001/jamanetworkopen.2025.15160)
Supplement: Supplement 1. — eFigure 1. Flowchart of Data Screening eFigure 2. Citation Network of Systematic Reviews with Heavy Citation Contamination eTable. DOIs of Systematic Reviews with Severe Citation Contamination and the DOIs of Their Cited Papers eAppendix 1. Python Source Code for Data Matching eAppendix 2. Full-Text Review Strategy [file jamanetwopen-e2515160-s001.pdf]

Supplemental Online Content

Tang G, Cai H. Citation contamination by paper mill articles in systematic reviews of the life sciences. *JAMA Netw Open.* 2025;8(6):e2515160. doi:10.1001/jamanetworkopen.2025.15160

eFigure 1. Flowchart of Data Screening

eFigure 2. Citation Network of Systematic Reviews with Heavy Citation Contamination

eTable. DOIs of Systematic Reviews with Severe Citation Contamination and the DOIs of Their Cited Papers

eAppendix 1. Python Source Code for Data Matching

eAppendix 2. Full-Text Review Strategy

This supplemental material has been provided by the authors to give readers additional information about their work.

© 2025 Tang G et al. *JAMA Network Open*.

eFigure 1. Flowchart of Data Screening

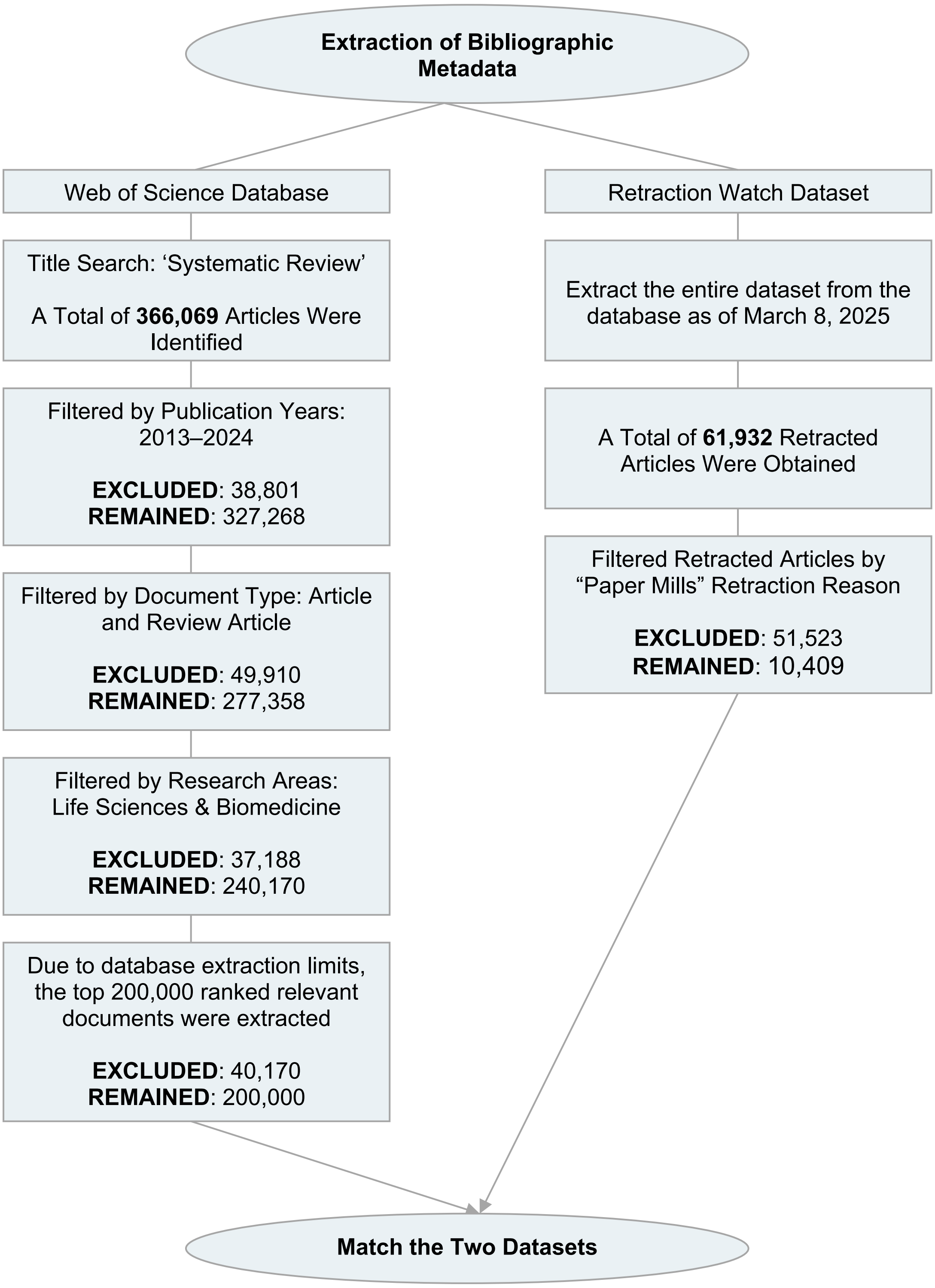

© 2025 Tang G et al. *JAMA Network Open*.

eFigure 2. Citation Network of Systematic Reviews with Heavy Citation Contamination

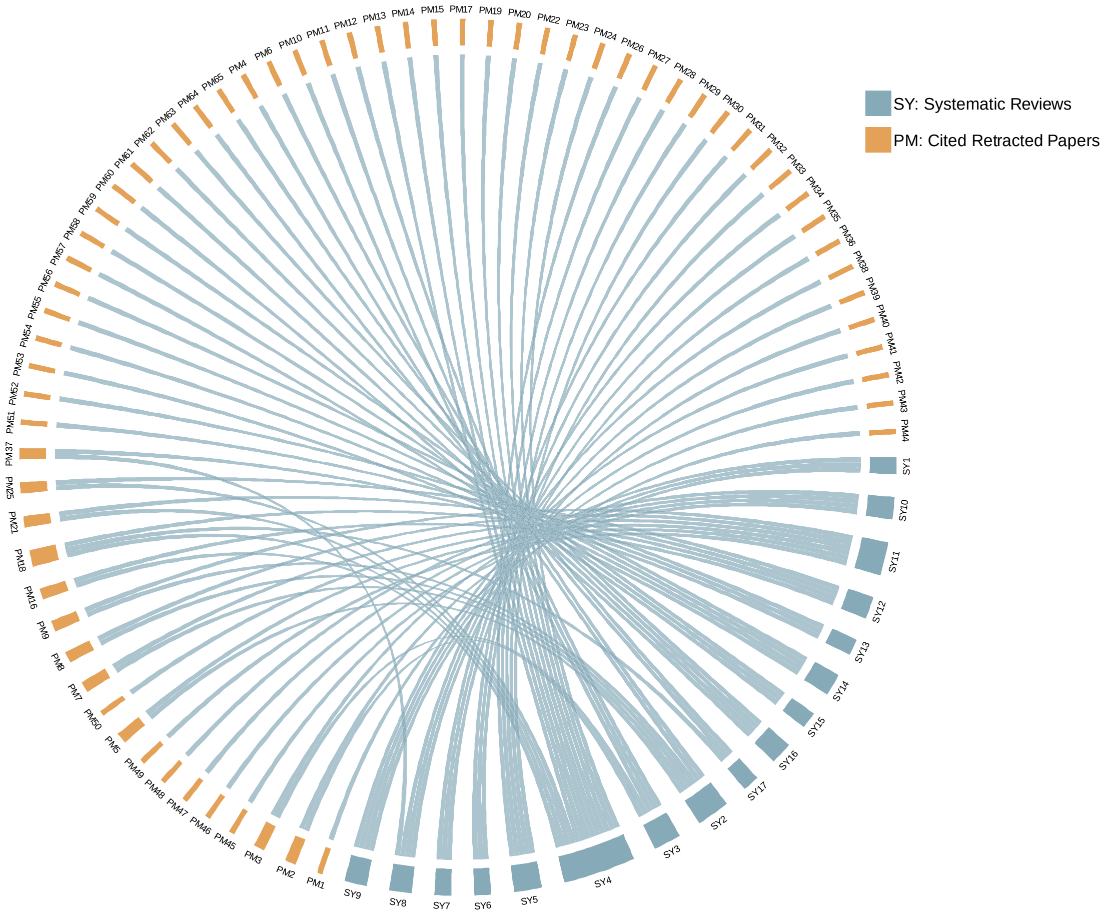

© 2025 Tang G et al. *JAMA Network Open*.

eTable. DOIs of Systematic Reviews with Severe Citation Contamination and the DOIs of Their Cited Papers

Systematic Review ID	DOI	Paper Mill Articles ID	DOI
SY1	10.3390/ijms22010321	PM1	10.1016/j.intimp.2017.11.038
SY1	10.3390/ijms22010321	PM2	10.1016/j.intimp.2020.106204
SY1	10.3390/ijms22010321	PM3	10.1016/j.lfs.2020.117849
SY2	10.3390/cancers13051137	PM4	10.1042/bsr20171546
SY2	10.3390/cancers13051137	PM5	10.3892/ijmm.2018.3853
SY2	10.3390/cancers13051137	PM6	10.3233/cbm-170388
SY2	10.3390/cancers13051137	PM7	10.1002/jcb.26763
SY2	10.3390/cancers13051137	PM8	10.3892/ol.2015.4001
SY2	10.3390/cancers13051137	PM9	10.1080/15384101.2019.1648958
SY3	10.3390/ijms23169354	PM3	10.1016/j.lfs.2020.117849
SY3	10.3390/ijms23169354	PM10	10.1016/j.biopha.2018.05.042
SY3	10.3390/ijms23169354	PM11	10.1186/s11671-021-03534-x
SY3	10.3390/ijms23169354	PM12	10.1016/j.intimp.2021.107691
SY3	10.3390/ijms23169354	PM2	10.1016/j.intimp.2020.106204
SY4	10.3390/cancers12113118	PM13	10.2147/ott.s201282
SY4	10.3390/cancers12113118	PM14	10.2147/ott.s213371
SY4	10.3390/cancers12113118	PM15	10.1002/jcp.29557
SY4	10.3390/cancers12113118	PM16	10.3892/ijo.2018.4615
SY4	10.3390/cancers12113118	PM17	10.1016/j.lfs.2020.117519
SY4	10.3390/cancers12113118	PM18	10.1002/ar.23940
SY4	10.3390/cancers12113118	PM19	10.3892/mmr.2019.9826
SY4	10.3390/cancers12113118	PM20	10.3892/mmr.2018.9750
SY4	10.3390/cancers12113118	PM21	10.1016/j.biopha.2018.05.078
SY4	10.3390/cancers12113118	PM22	10.1002/jcb.27108
SY4	10.3390/cancers12113118	PM23	10.1016/j.biopha.2018.03.043
SY4	10.3390/cancers12113118	PM24	10.1080/15384101.2020.1749466
SY4	10.3390/cancers12113118	PM25	10.1016/j.biopha.2018.03.122
SY5	10.3389/fphar.2022.879704	PM26	10.1016/j.omtn.2020.09.020
SY5	10.3389/fphar.2022.879704	PM27	10.18632/oncotarget.23416
SY5	10.3389/fphar.2022.879704	PM28	10.1002/jcp.28902
SY5	10.3389/fphar.2022.879704	PM29	10.1016/j.omtn.2018.12.006
SY5	10.3389/fphar.2022.879704	PM30	10.2147/cmar.s332640
SY6	10.1002/jcp.28782	PM31	10.3892/mmr.2017.6841
SY6	10.1002/jcp.28782	PM32	10.1002/jcb.26141
SY6	10.1002/jcp.28782	PM33	10.3892/or.2017.5810
SY7	10.1108/jadee-12-2022-0262	PM34	10.1080/09064710.2020.1840618
SY7	10.1108/jadee-12-2022-0262	PM35	10.1080/09064710.2021.1890202
SY7	10.1108/jadee-12-2022-0262	PM36	10.1080/09064710.2021.2005131
SY8	10.1007/s00432-023-05273-1	PM37	10.1186/s13046-018-0845-9
SY8	10.1007/s00432-023-05273-1	PM38	10.1016/j.lfs.2021.119495
SY8	10.1007/s00432-023-05273-1	PM39	10.1016/j.omtn.2019.10.047
SY8	10.1007/s00432-023-05273-1	PM40	10.1016/j.omtn.2019.04.030
SY9	10.1080/14737159.2021.1873131	PM41	10.1002/jcb.28409

© 2025 Tang G et al. *JAMA Network Open*.

Systematic Review ID	DOI	Paper Mill Articles ID	DOI
SY9	10.1080/14737159.2021.1873131	PM42	10.1039/c8ra05724a
SY9	10.1080/14737159.2021.1873131	PM43	10.1016/j.intimp.2018.03.023
SY9	10.1080/14737159.2021.1873131	PM44	10.1016/j.biopha.2018.05.069
SY10	10.1007/s10585-019-10013-2	PM45	10.3892/mmr.2017.8361
SY10	10.1007/s10585-019-10013-2	PM46	10.1371/journal.pone.0127682
SY10	10.1007/s10585-019-10013-2	PM47	10.3892/mmr.2018.9292
SY10	10.1007/s10585-019-10013-2	PM48	10.3892/ol.2016.4577
SY11	10.3390/cancers13143393	PM49	10.1080/15384101.2018.1475829
SY11	10.3390/cancers13143393	PM5	10.3892/ijmm.2018.3853
SY11	10.3390/cancers13143393	PM50	10.3892/mmr.2014.2123
SY11	10.3390/cancers13143393	PM7	10.1002/jcb.26763
SY11	10.3390/cancers13143393	PM8	10.3892/ol.2015.4001
SY11	10.3390/cancers13143393	PM9	10.1080/15384101.2019.1648958
SY12	10.3389/fsurg.2020.00043	PM21	10.1016/j.biopha.2018.05.078
SY12	10.3389/fsurg.2020.00043	PM18	10.1002/ar.23940
SY12	10.3389/fsurg.2020.00043	PM16	10.3892/ijo.2018.4615
SY12	10.3389/fsurg.2020.00043	PM25	10.1016/j.biopha.2018.03.122
SY13	10.1155/2020/6786875	PM51	10.3892/ijo.2018.4412
SY13	10.1155/2020/6786875	PM52	10.3892/ijo.2018.4467
SY13	10.1155/2020/6786875	PM37	10.1186/s13046-018-0845-9
SY14	10.3389/fcell.2024.1284934	PM53	10.1080/21691401.2019.1640233
SY14	10.3389/fcell.2024.1284934	PM54	10.26355/eurrev_202002_20153
SY14	10.3389/fcell.2024.1284934	PM55	10.1002/jcb.27676
SY14	10.3389/fcell.2024.1284934	PM56	10.1016/j.lfs.2020.117672
SY15	10.3390/ijms241310456	PM57	10.1155/2021/4883509
SY15	10.3390/ijms241310456	PM58	10.1155/2021/3957738
SY15	10.3390/ijms241310456	PM59	10.1186/s13046-019-1070-x
SY16	10.3390/ijms20061463	PM60	10.1159/000493827
SY16	10.3390/ijms20061463	PM61	10.1016/j.biopha.2018.04.191
SY16	10.3390/ijms20061463	PM62	10.1016/j.biopha.2018.03.040
SY16	10.3390/ijms20061463	PM63	10.1016/j.biopha.2018.06.127
SY17	10.1186/s12967-020-02644-x	PM64	10.3892/ijo.2015.3112
SY17	10.1186/s12967-020-02644-x	PM18	10.1002/ar.23940
SY17	10.1186/s12967-020-02644-x	PM65	10.1042/bsr20160521

© 2025 Tang G et al. *JAMA Network Open*.

eAppendix 1. Python Source Code for Data Matching

We developed a Python script to perform citation matching. The script extracts DOIs from the reference fields in the original Web of Science database and matches them against entries in the Retraction Watch dataset:

```
import re
import pandas as pd

# File Path
wos_file = "File Path"
retracted_file = "File Path"

# Parsing WOS data functions
def parse_wos_data(file_path):
    wos_articles = []
    current_article = {}
    with open(file_path, 'r', encoding='utf-8') as file:
        for line in file:
            line = line.strip()

            if line.startswith("PT "):
                if current_article:
                    wos_articles.append(current_article)
                current_article = {}

            elif line.startswith("TI "):
                current_article["Title"] = line[3:].strip()

            elif line.startswith("AU "):
                current_article["Authors"] = line[3:].strip()

            elif line.startswith("SO "):
                current_article["Journal"] = line[3:].strip()

            elif line.startswith("CR "):
                current_article["References"] = []
                while True:
                    line = file.readline().strip()
                    if line.startswith("NR") or not line:
                        break
                    dois = re.findall(r'DOI\s([^\s,;]+)', line)
                    current_article["References"].extend(dois)

            elif line.startswith("PY "):
                current_article["PublicationYear"] = line[3:].strip()

            elif line.startswith("PD "):
                current_article["PublicationDate"] = line[3:].strip()

            elif line.startswith("EY "):
                current_article["EarlyAccessYear"] = line[3:].strip()

            elif line.startswith("EA "):
                current_article["EarlyAccessDate"] = line[3:].strip()
```

© 2025 Tang G et al. *JAMA Network Open*.

```
            elif line.startswith("DI "):
                current_article["DOI"] = line[3:].strip().lower()

            elif line.startswith("SC "):
                current_article["Subject"] = line[3:].strip()

        if current_article:
            wos_articles.append(current_article)

    return pd.DataFrame(wos_articles)

# Loading WOS data
wos_data = parse_wos_data(wos_file)

wos_data["PublicationYear"] = wos_data.apply(
    lambda row: row["EarlyAccessYear"] if pd.notna(row.get("EarlyAccessYear")) else row["PublicationYear"], axis=1
)

wos_data["PublicationDate"] = wos_data.apply(
    lambda row: row["EarlyAccessDate"] if pd.notna(row.get("EarlyAccessDate")) else row["PublicationDate"], axis=1
)

# Filling missing values
wos_data["References"] = wos_data["References"].apply(lambda x: x if isinstance(x, list) else [])
wos_data.fillna("Unknown", inplace=True)

# Loading Retraction Watch dataset
retracted_data = pd.read_csv(retracted_file)
retracted_data["OriginalPaperDOI"] = retracted_data["OriginalPaperDOI"].str.strip().str.lower()

# Extract all DOIs in WOS
wos_dois = [doi.lower() for references in wos_data["References"] if references for doi in references]

# Check DOI intersection
matched_dois = set(wos_dois).intersection(set(retracted_data["OriginalPaperDOI"]))

# Constructing matching result dataset
matched_records = []
for _, row in wos_data.iterrows():
    references = [doi.lower() for doi in row["References"]]
    matches = set(references).intersection(matched_dois)
    for match in matches:
        retraction_info = retracted_data.loc[retracted_data["OriginalPaperDOI"] == match]
        for _, ret_row in retraction_info.iterrows():
            matched_records.append({
                "WOS Title": row.get("Title", ""),
                "WOS Authors": row.get("Authors", ""),
                "WOS Journal": row.get("Journal", ""),
                "PublicationYear": row.get("PublicationYear", "Unknown"),
                "PublicationDate": row.get("PublicationDate", "Unknown"),
                "Subject": row.get("Subject", "Unknown"),
                "WOS DOI": row.get("DOI", ""),
                "Matched DOI": match,
                "Original Paper Date": ret_row["OriginalPaperDate"],
                "Retraction Date": ret_row["RetractionDate"],
            })
```

© 2025 Tang G et al. *JAMA Network Open*.

```
matched_df = pd.DataFrame(matched_records)

# Deduplication
matched_df.drop_duplicates(inplace=True)

# Output matching results
if not matched_df.empty:
    output_file = "file path"
    matched_df.to_csv(output_file, index=False)
    print(f"The matching results have been saved as: {output_file}")
else:
    print("No records were found that cite any retracted articles");
```

© 2025 Tang G et al. *JAMA Network Open*.

eAppendix 2. Full-Text Review Strategy

To accurately determine whether systematic reviews had cited and incorporated paper mill articles, we conducted a comprehensive full-text review of all records retained after the initial screening. The objective of this step was to examine how the potentially contaminated articles were used—whether they were included in evidence synthesis, discussed narratively, or cited peripherally (e.g., in background or references).

The full-text screening process was carried out independently by two researchers (Gengyan Tang and Hao Cai) between March 17 and March 21, 2025. Each reviewer downloaded and read the complete version of each systematic review. For the majority of articles, access was provided through the institutional subscriptions available via the University of Calgary Library. For publications that were not available through this channel—particularly those from smaller publishers or regional journals—full-text access was obtained with the assistance of librarians at Chongqing Medical University Library, who helped retrieve specific articles upon request.

The review followed a standardized three-step process to determine the presence and significance of any citation of paper mill articles:

(1) DOI-based identification: For each matched paper mill article, we first used the PDF full-text search function to locate its corresponding reference number or author name within the reference list of the systematic review.

(2) In-text tracing: Next, we used the same search function to trace where and how this reference number appeared within the main text of the review.

(3) Contextual analysis: Upon locating the in-text citations, we read the relevant sections to determine whether the cited article was used in the evidence table, evidence synthesis, or in the narrative justification of findings. If so, we classified the systematic review as contaminated, since such citations could potentially bias the conclusions of the review. In contrast, if the citation appeared only in the background, methodology description, or discussion section, the systematic review was not considered contaminated, as the potential influence on its findings was judged to be minimal.

To ensure consistency and reliability, Gengyan Tang and Hao Cai employed a standardized data extraction form throughout the review process. Any discrepancies or disagreements were resolved through discussion and consensus.

This rigorous full-text reviewing and contextual evaluation strategy allowed us to categorize the level and impact of contamination with greater precision, strengthening the robustness and interpretability of our findings.

© 2025 Tang G et al. *JAMA Network Open.*
