# Supplementary material for: Citation Contamination by Paper Mill Articles in Systematic Reviews of the Life Sciences
Source: JAMA Netw Open. 2025 Jun 12;8(6):e2515160. doi: 10.1001/jamanetworkopen.2025.15160 (PMC12163679; doi:10.1001/jamanetworkopen.2025.15160)
Supplement: Supplement 2. — Data Sharing Statement [file jamanetwopen-e2515160-s002.pdf]

Data Sharing Statement

Tang. Citation Contamination by Paper Mill Articles in Systematic Reviews of the Life Sciences. *JAMA Netw Open*. Published June 12, 2025.
doi:10.1001/jamanetworkopen.2025.15160

Data
Data available: Yes
Data types: Data (not involving human participants)
How to access data: The data that support the findings of this study are openly available in Open Science Framework (OSF) at: https://osf.io/7s9dy/
When available: With publication

Supporting Documents
Document types: None

Additional Information
Who can access the data: Anyone requesting the data
Types of analyses: The data will be made available for analyses aimed at replicating or validating the findings of this study
Mechanisms of data availability: with investigator support
